# Genome-Wide Approaches to Dissect the Roles of RNA Binding Proteins in Translational Control: Implications for Neurological Diseases

**DOI:** 10.3389/fnins.2012.00144

**Published:** 2012-10-02

**Authors:** Katannya Kapeli, Gene W. Yeo

**Affiliations:** ^1^Department of Cellular and Molecular Medicine, University of California San DiegoLa Jolla, CA, USA; ^2^UCSD Stem Cell Program, University of California San DiegoLa Jolla, CA, USA; ^3^UCSD Moores Cancer Center, University of California San DiegoLa Jolla, CA, USA; ^4^Institute for Genomic Medicine, University of California San DiegoLa Jolla, CA, USA

**Keywords:** translation, neurological disease, RNA binding proteins, ribosome profiling, CLIP

## Abstract

Translational control of messenger RNAs (mRNAs) is a key aspect of neurobiology, defects of which can lead to neurological diseases. In response to stimuli, local translation of mRNAs is activated at synapses to facilitate long-lasting forms of synaptic plasticity, the cellular basis for learning, and memory formation. Translation, as well as all other aspects of RNA metabolism, is controlled in part by RNA binding proteins (RBPs) that directly interact with mRNAs to form mRNA-protein complexes. Disruption of RBP function is becoming widely recognized as a major cause of neurological diseases. Thus understanding the mechanisms that govern the interplay between translation control and RBP regulation in both normal and diseased neurons will provide new opportunities for novel diagnostics and therapeutic intervention. As a means of studying translational control, genome-wide methods are emerging as powerful tools that have already begun to unveil mechanisms that are missed by single-gene studies. Here, we describe the roles of RBPs in translational control, review genome-wide approaches to examine translational control, and discuss how the application of these approaches may provide mechanistic insight into the pathogenic underpinnings of RBPs in neurological diseases.

## Introduction

Analogous to DNA, which is organized and packed via strong associations with histones in the nucleus, precursor, and mature messenger RNAs (mRNAs) never exist as “naked” ribonucleic acid sequences. After transcription in the nucleus, RNA binding proteins (RBPs) recognize *cis*-regulatory RNA elements within precursor mRNA sequence to form messenger ribonucleoprotein (mRNP) complexes. Again, analogous to DNA-binding proteins such as transcription factors that regulate gene expression by binding to DNA elements in the promoters of genes, RBPs regulate the fate of target RNAs by interacting with specific sequences or RNA secondary structural features within the transcribed RNA molecule. These *cis*-regulatory RNA elements can be found in the 5′ and 3′ untranslated regions (UTRs), introns, and exons of all protein-coding genes. RNA elements in 5′ and 3′ UTRs are frequently involved in targeting RNA to specific cellular compartments, affecting 3′ end formation, controlling RNA stability, and regulating mRNA translation. RNA elements in introns and exons are known to function as splicing enhancers or silencers to control the process of precursor mRNA splicing (Jensen et al., 2009).

A genome-wide survey of 323 mouse RBPs by *in situ* hybridization in the developing brain yielded the surprising result that two-thirds of those RBPs are expressed in a cell type specific manner (McKee et al., 2005). Compared to other cells in the body, the complex structure and specialization of neurons explains the need for having many RBPs to maintain proper neural function. Consistent with the crucial roles of RBPs in regulating RNA homeostasis in the nervous system, mutations that impair RBP function have been linked to severe neurological diseases such as Fragile X syndrome (FXS), Fragile X-associated tremor/ataxia syndrome (FXTAS), Amyotrophic lateral sclerosis (ALS), Frontotemporal lobar dementia (FTLD), Spinal muscular atrophy (SMA), and Myotonic dystrophy (Lukong et al., 2008). To understand the impact of mutations within RBPs in neurodegeneration, we need to elucidate the normal activities of RBPs in neurons. It is well-known that RBPs are intimately involved with the regulation of alternative splicing, a process by which numerous isoforms are generated from a single genetic loci, and is in fact, more prevalent in the nervous system than in any other cell types (Yeo et al., 2004; Wang et al., 2008a). RBPs are required to protect mRNAs during their transport from the soma to distal axonal and dendritic locations, and once at these locations, RBPs mediate local *de novo* synthesis of proteins (translation). Local translation at or near axonal and dendritic synapses is the underlying mechanism of synaptic plasticity (Sutton and Schuman, 2006), which refers to the ability of synapses to undergo long-lasting biochemical and morphological changes in response to stimuli (Richter and Klann, 2009). As a result, local translation is critical for cognition and memory. Local synaptic translation is also critical for axon guidance and nerve regeneration (Willis et al., 2005). Accordingly, pharmacological inhibition of protein synthesis prevents some forms of synaptic plasticity in cultured neurons and attenuates long-term memory in mice (Scharf et al., 2002; Kelleher et al., 2004; Banko et al., 2005; Sutton and Schuman, 2006).

Given the significance of RBP biology and mRNA translation in controlling neuron structure and function, advances in sequencing and microarray technology have sparked the development of genome-wide methods that enable the neuroscience community to dissect the roles that RBPs play in controlling mRNA translation in the brain. Here we review how RBPs associate with different mRNP complexes to regulate translation, summarize emerging genome-wide methods that enable an unbiased examination of translation on a global scale, and discuss how genome-wide studies using these methods have and will continue to aid our understanding of translational control in normal and pathological neurobiology.

## Messenger RNP Complexes and Translational Control

**Figure 1:**
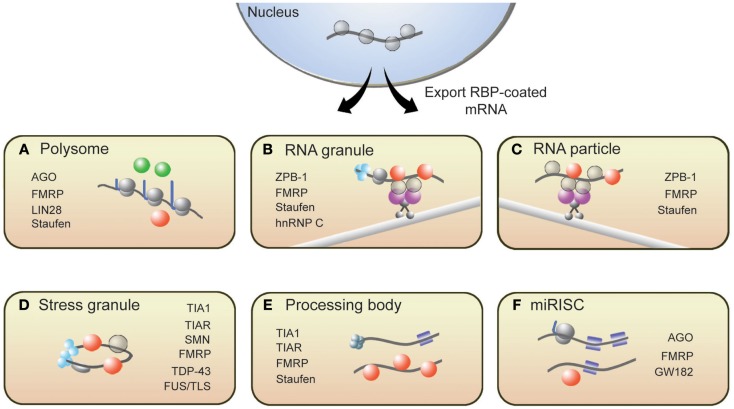
**Messenger RNAs associate with several RNP structures that influence their translational state**. **(A)** Polysomes, sites of translation, contain RBPs that activate (green spheres) or repress (red spheres) translation. Following synthesis and processing, mRNA is exported from the nucleus and transported throughout the cell along microtubules via **(B)** RNA granules and **(C)** RNA particles. Repressor RBPs (red spheres) are present within RNA particles to ensure that mRNAs are not translated during transit. Messenger RNAs within RNA granules are associated with translation initiation machinery (light blue spheres) including ribosomes, suggesting that translation has commenced but is halted during transit. The translational fate of mRNA is dictated in part by the RBPs bound to them. If targeted by repressor RBPs or miRISCs (blue squares), mRNAs will associate with **(D)** stress granules, **(E)** processing bodies, or **(F)** miRISC structures resulting in either degradation or translational repression. Some RBPs present in neuronal RNP complexes are listed.

From synthesis to destruction, mRNAs are coated with RBPs that sequester mRNA into mRNP complexes and ultimately influence their cellular fate. These mRNP complexes, as depicted in Figure 1, are polysomes, RNA granules, RNA particles, stress granules (SGs), processing bodies (P-bodies), and RNA-induced silencing complexes (RISCs). This section provides a brief description of these complexes and introduces RBPs with roles in translation that associate with these complexes (for more details, see Kiebler and Bassell, 2006; Sossin and DesGroseillers, 2006; Erickson and Lykke-Andersen, 2011).

Polysome complexes are the centers of protein production and are present in the cell body, axon, and dendrites of a neuron (Steward and Levy, 1982; Giuditta et al., 2002). RBPs such as Lin28 have been shown to decorate polysomes and promote translation (Balzer and Moss, 2007). It is important to note, however, that polysome-associated mRNAs can be translationally repressed. Several groups have reported instances where translational inhibition of certain proteins did not correspond to a decrease in ribosome number on the encoding mRNAs (Olsen and Ambros, 1999; Braat et al., 2004; Nottrott et al., 2006; Petersen et al., 2006). Such observations likely captured an event called ribosomal stalling, where ribosomes temporarily or permanently stop elongating along transcripts. The RBPs FMRP and Staufen have been shown to induce ribosomal stalling (Thomas et al., 2009; Darnell et al., 2011). MicroRNA-loaded RISCs (miRISCs), which also associate with polysomes, can repress translation by promoting ribosomal pausing (Maroney et al., 2006; Nottrott et al., 2006; Petersen et al., 2006).

For local translation to occur at synapses, mRNAs must be transported from the soma to synapses. RNA particles and RNA granules function to traffic mRNAs to designated subcellular compartments. These structures are complexes of mRNAs and interacting RBPs, motor proteins, and adaptor proteins that tether the RBPs to motor complexes. While in transit, transcripts are both protected from degradation and are translationally repressed until the appropriate signals are received. The RBP Zip-code binding protein 1 (ZBP1) is a well-known regulator of mRNA transport and translational repression (Huttelmaier et al., 2005). Other RBPs such as Staufen, FMRP, and Pumilio also suppress translation of transcripts in RNA particles and RNA granules (Kiebler and DesGroseillers, 2000; Wang et al., 2010). An important distinction between these two transport complexes is that RNA granules contain ribosomes while RNA particles do not (Sossin and DesGroseillers, 2006). The presence of ribosomes within RNA granules suggests that the translation of associated mRNAs is blocked at the step of translational elongation.

RNA binding proteins are present within several other mRNP complexes that contain translationally repressed mRNAs. In response to cellular stress, TIA1, TIAR, G3BP, and other RBPs aggregate to form SGs, which contain untranslated mRNAs (Buchan and Parker, 2009; Kedersha and Anderson, 2009). SGs are proposed to safeguard specific mRNAs from destruction during cellular stress and, upon relief of the stress signal, disassemble to allow translationally repressed mRNAs to re-enter translation. P-bodies are another type of mRNP structure containing non-translating mRNAs that are destined for degradation (Coller and Parker, 2005; Teixeira et al., 2005) or are eventually released to re-associate with polysomes (Brengues et al., 2005). While the composition of P-bodies is not fully characterized, they generally contain decapping enzymes, exonucleases, translational repressors, microRNA (miRNA) silencing machinery, and translation-regulating RBPs, including CPEB, Staufen, and eIF4E (Parker and Sheth, 2007). Lastly, guided by miRNAs, RISCs repress translation of target mRNAs at the stage of translation initiation or elongation (Valencia-Sanchez et al., 2006). RBPs such as FMRP have been shown to associate with miRISC (Caudy et al., 2002; Witold, 2005) and can either promote or antagonize the repressive actions of miRISC (Brodersen and Voinnet, 2009). These translation-silencing mRNPs have been observed to interact with one another, and some components of SGs overlap with P-bodies (Kedersha et al., 2005) and miRISCs with P-bodies (Liu et al., 2005; Edbauer et al., 2010); however, the mechanisms that mediate these interactions remain to be established.

The interactions between RBPs and mRNAs are dynamic, allowing mRNAs to move from one mRNP to another in a controlled, bidirectional manner. This is an important feature of mRNA regulation because it ensures that the post-transcriptional fate of mRNAs is responsive to intracellular and extracellular signals. Signaling pathways, which are stimulated by various intracellular and extracellular cues, largely influence the mRNP distribution, and thus translational status, of mRNAs by regulating the expression and/or function of RBPs. For example, activation of the mTOR signaling results in the phosphorylation of FMRP (Narayanan et al., 2008); this post-translational modification affects the ability of FMRP to regulate translation (Ceman et al., 2003) and associate with RISC complexes (Cheever and Ceman, 2009). In response to signal-induced synaptic activation, the RBP Staufen was shown to activate translation by redistributing target mRNAs from RNA granules to translating polyribosomes (Krichevsky and Kosik, 2001).

## Genome-Wide Approaches to Study Translation

Advancements in technologies have significantly improved our ability to study translation at a genome-wide scale. Highly parallel techniques such as microarrays and high-throughput sequencing (deep sequencing) have revolutionized approaches to gene discovery, offering unbiased approaches and may be modified to investigate specific aspects of RNA regulation. In this section, we highlight studies that have utilized such technologies to investigate translational control at the genome-wide level both within neural and non-neural contexts (summarized in Table 1).

**Table 1:** **Genome-wide methods to study translation**.

	RNA isolation methodology	Novelty/advantages	Limitations	Reference
Polysome profiling	Purification of polysome-associated mRNAs by centrifugation through a sucrose gradient	Original method to examine translation status of transcriptome	Labor intensive; scaling issues; does not differentiate between active and stalled ribosomes	Zong et al. (1999)
TRAP	Immunoprecipitation (IP) of EFGP-L10a-associated mRNAs from mouse brain tissue	Examines polysome-associated mRNAs within a specific cell type *in vivo*	Each bacTRAP mouse line is limited to surveying one cell type; EGFP antibodies are costly relative to anti-HA antibody; does not differentiate between active and stalled ribosomes	Heiman et al. (2008), Doyle et al. (2008)
RiboTag	IP of Rlp22-HA-associated mRNAs from mouse tissue	Examines polysome-associated mRNAs within a specific cell type *in vivo*; takes advantage of Cre recombinase-expressing mouse lines to expand the range of cell types that can be investigated; commercial anti-HA antibody is less costly than in-house EGFP (see TRAP)	Does not differentiate between active and stalled ribosomes	Sanz et al. (2009)
Ribosome profiling	Nuclease digestion of polysome complexes, followed by centrifugation through a sucrose gradient or cushion to purify ribosome-mRNA complexes; ribosome-protected fragments are deep sequenced	Determines ribosome position and translation efficiency for individual mRNAs; reveals novel translational regulatory features (e.g., uORFs, start and termination sites, ribosome stall position)	May be difficult to apply to mouse models	Ingolia et al. (2009), Ingolia et al. (2011)
CLIP	UV-mediated crosslinking of mRNA-protein complexes, followed by nuclease digestion and IP of RBP of interest to recover RBP-protected mRNA fragments	Demonstrated the feasibility of crosslinking mRNA and protein using UV irradiation, which results in covalent bonds	Generated a limited dataset with a high false positive rate; low crosslinking efficiency	Ule et al. (2003)
CLIP-seq or HITS-CLIP	CLIP coupled with deep sequencing	Identifies direct RBP binding sites at nucleotide resolution	Low crosslinking efficiency	Licatalosi et al. (2008)
iCLIP	HITS-CLIP with modifications whereby a 5′ adapter and random barcode is attached to cDNA molecules after reverse transcription; the former modification allows for circularization of the cDNA	Introduction of a random barcode enables identification and quantification of unique cDNA products; cDNA circularization allows for the capture and sequencing of truncated cDNAs usually lost with standard CLIP, revealing crosslinking sites at nucleotide resolution	Low crosslinking efficiency	König et al. (2010)
PAR-CLIP	Photoreactive ribonucleoside analogs (e.g., 4SU or 6-SG) are incorporated into mRNA; nuclease digestion and IP of RBP of interest isolates RBP-protected mRNA fragments	Use of 4SU or 6-SG increases crosslinking efficiency; exact crosslinking sites are revealed after sequencing by T to C transitions in the cDNA prepared from RBP-bound mRNA	Some RBPs may not be amenable to PAR-CLIP	Hafner et al. (2010), Castello et al. (2012)
iPAR-CLIP	PAR-CLIP method applied to *C. elegans* exposed to 4SU	First demonstration of CLIP in a non-cell line system; allows for physiologically relevant, context-dependent studies of protein-RNA interactions in *C. elegans*	Technique yet to be applied to other *in vivo* models	Jungkamp et al. (2011)

### Gene expression profiling using microarrays

With the ability to examine gene expression on a global scale, microarray studies have provided evidence that diverse populations of mRNAs are localized at synapses. Martin and colleagues (Poon et al., 2006) were one of the first groups to examine synaptically localized mRNAs in rat neurons by mechanically separating axonal and dendritic processes from the cell body and performing microarray analysis on the isolated mRNA. Strikingly, they found that a significant proportion of synaptic mRNAs encoded translation factors and regulators, and proposed that this may be a general mechanism to enhance the capacity for local translation at synapses. Zhong et al. (2006) performed microarray studies on rat brain mRNA, which led to the discovery that the repertoire of synaptic mRNAs is more diverse than previously thought. The group not only identified transcripts that encoded translation factors and regulators, but also transcripts that encoded receptor and channel proteins, signaling molecules, cytoskeleton, and adhesion proteins, membrane trafficking proteins, and molecules involved in protein degradation. Additional studies have examined the synaptic transcriptome within other contexts, such as brain-derived nerve growth stimulation (Schratt et al., 2004) or neurons displaying molecular signatures of Alzheimer’s disease (Williams et al., 2009). Interestingly, results from these and other microarray studies displayed little overlap, suggesting that a large number of mRNAs can be sequestered at synapses but that their localization largely depends on the cellular context.

### Polysome profiling

**Figure 2:**
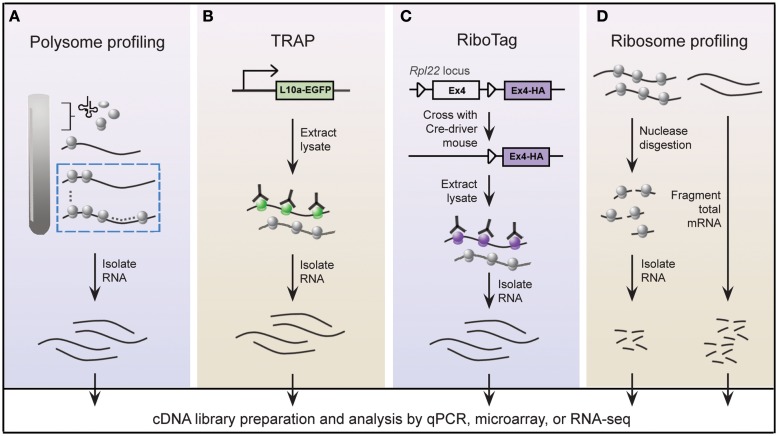
**Schematic of genome-wide methods to study polysome-associated mRNAs *in vitro* and *in vivo***. **(A)** With polysome profiling, cytoplasmic lysates from cells are layered onto a sucrose gradient and undergo centrifugation to separate tRNAs, 40S, 60S, and 80S ribosomes, and polysomes. Messenger RNAs from fractions corresponding to polysomes (dashed blue box) are isolated and identified by various approaches. **(B)** Engineered bacTRAP mice drive expression of EGFP-tagged L10a, a ribosomal protein found in polysomes (green ribosomes), from promoters that are activated in specific cells of the central nervous system. EGFP-L10a-mRNA complexes are immunopurified from brain tissue from bacTRAP mice, and associated mRNAs are identified by various techniques. **(C)** The RiboTag mouse carries an Rpl22 allele with a floxed wild-type C-terminal Exon4 followed by a HA-tagged Exon4. When the RiboTag mouse is crossed with a mouse expressing Cre-recombinase in a cell-type specific manner, Cre-recombinase activates expression of HA-tagged Rpl22, which incorporates into polysomes (purple ribosomes). Homogenized tissues from the offspring are subjected to co-immunoprecipitation using antibodies against HA, and associated mRNAs are identified by various techniques. **(D)** Using ribosome profiling to identify ribosome occupancy on mRNAs, cycloheximide-treated lysates from cultured cells are digested by micrococcal nucleases to remove mRNA sequences that are not bound by ribosomes (left). The resulting monosome complexes are purified by ultracentrifugation through a sucrose gradient or cushion. Ribosome-protected fragments are recovered and deep sequenced. In parallel, total mRNA from a similar preparation of cycloheximide-treated lysate is fragmented and deep sequenced (right), and serves as a normalizing control.

A widely held view is that mRNA expression correlates closely with expression of the protein it encodes; this is certainly true in most instances, but is not always the case (Anderson and Seilhamer, 1997; Gygi et al., 1999). Indeed, the lack of correlation between mRNA and protein expression is expected given that multiple mechanisms are in place to control translation of mRNA. In this regard, microarray studies provide limited insight into the translational status of mRNA. An alternative approach called polysome profiling exploits the observation that, in general, polysome-associated mRNAs are translationally active. By separating polysome, monosome, and other mRNP complexes by centrifugation through a sucrose gradient, well-translated mRNAs can easily be distinguished from poorly translated mRNAs (Figure 2A). Morris and colleagues were one of the pioneering groups that used polysome profiling to examine the translation state of the transcriptome (Zong et al., 1999). Specifically, total cytoplasmic extracts from cultured human fibroblasts were layered onto sucrose gradients and centrifuged to separate the different mRNP complexes. Transcripts residing in high-density fractions containing polysome species were examined by microarray analysis to identify well-translated mRNAs (Figure 2A), while transcripts sequestered within the low-density fractions were also examined to identify poorly translated mRNAs.

Polysome profiling has been readily applied to various cellular contexts and cell types, including neurons (Johannes et al., 1999; Preiss et al., 2003; Rajasekhar et al., 2003; Schratt et al., 2004; Iguchi et al., 2006). A study that nicely demonstrated the utility of polysome profiling examined the widespread inhibition of translation in response to cellular stress, a process that appears to involve movement of mRNAs from polysome complexes to P-bodies. The movement of transcripts from polysomes to P-bodies is thought to be a general phenomenon in eukaryotic cells, yet evidence to support this view is based on single-gene studies. To address whether this view is in fact a general phenomenon or limited to a subset of mRNAs, Arribere et al. (2011) used polysome profiling in combination with translation inhibitors to measure the translational activity and ribosome occupancy upon glucose withdrawal and at different times following glucose re-addition. This study illustrated the power of genome-wide studies: Arribere and colleagues were able to provide mechanistic insight to translational control based on an examination of the entire transcriptome, not just representative transcripts. Notably, they showed that a substantial portion of mRNAs, many of which encode survival factors, is actively translated during stress; this finding disputed the prevailing notion that translational inhibition is widespread during stress. They also found that re-entry of pre-existing mRNAs, presumably from P-bodies, into polysomes is restricted to a subset of mRNAs rather than a general phenomenon as initially proposed (Brengues et al., 2005; Teixeira et al., 2005; Brengues and Parker, 2007; Hoyle et al., 2007).

As with any technique, polysome profiling has several limitations. From a technical perspective, this technique is labor intensive and difficult to scale-up (Larsson and Nadon, 2008). Furthermore, polysome profiling should be performed with uniform cell populations due to the heterogenic nature of transcriptomes between cell types; this presents a significant challenge in performing polysome profiling *in vivo*. A final consideration concerns the underlying assumption of the technique that mRNAs bound by multiple ribosomes are translationally active. Both active and stalled ribosomes have been shown to co-sediment during isolation of polysome complexes through sucrose gradients (Sivan et al., 2007), indicating that polysome profiling does not completely distinguish translationally active from repressed mRNAs. Complementary molecular studies that directly measure *de novo* protein synthesis would be useful to discriminate translating from non-translating polysome-associated mRNAs. Despite these limitations, polysome profiling has been extremely successful in identifying translationally active mRNAs.

### Targeting ribosomes: Immunoprecipitation-based methods to isolate polysomes *in vivo*

Gene expression patterns vary greatly between cell types, thereby requiring that genome-wide studies be performed using homogeneous cell populations. The difficulty of performing polysome profiling *in vivo* is due to the challenge of extracting homogeneous cell populations in sufficient quantities without affecting the transcriptome (Okaty et al., 2011). The mammalian brain is an especially challenging model because of its immense heterogeneity. Enrichment methods, such as fluorescence-activated cell sorting or laser-capture microdissection, accurately separate genetically and/or morphologically distinct cells; however, manipulation of purified cells during isolation steps may alter the transcriptional profile (Okaty et al., 2011). These challenges must be addressed in order to achieve the full benefits of this powerful technique *in vivo*, giving us a better understanding of translational control within a physiologically relevant setting. Two groups independently tackled this issue by genetically altering mice to express epitope-tagged versions of ribosome protein subunits (Heiman et al., 2008; Sanz et al., 2009). All translated mRNAs are, at one point, associated with ribosomes; thus affinity purification of epitope-tagged ribosomes would allow for isolation of polysome-associated mRNAs. Heiman and colleagues termed their methodology translating ribosome affinity purification, or TRAP, which involves a series of bacterial artificial chromosome transgenic mice, called bacTRAP mice. In these genetically modified mice, expression of EGFP-tagged ribosomal protein L10a is driven by defined promoters that are activated in specific cell types of the central nervous system (CNS; Figure 2B; Heiman et al., 2008). Heiman and colleagues used bacTRAP mice that expressed EGFP-L10a from the Drd1a receptor or Drd2 receptor to isolate polysome-associated mRNA from striatonigral or striatopallidal cells, respectively, of the mouse striatum. Cells derived from the brain tissue of these mice were lysed in cycloheximide-spiked buffer to halt elongating ribosomes. Polysome-associated RNA corresponding to the specific cell type was isolated by immunoprecipitating for EGFP-L10a and examined by microarray analysis. The group demonstrated the power of TRAP technology by identifying polysome-associated mRNAs unique to four neuronal populations that are intermixed and morphologically indistinguishable. In an accompanying paper, Doyle et al. (2008) demonstrated the generality of TRAP with a comprehensive study of polysome profiles for 24 additional CNS cell types.

Using a strategy similar to TRAP, Sanz and colleagues engineered a mouse line called RiboTag that contains three HA tags inserted into the locus of *Rlp22*, a gene that encodes a ribosome protein present within polysomes (Figure 2C; Sanz et al., 2009). Expression of the *Rlp22^HA^* allele depends on Cre recombination such that in the absence of Cre recombinase only endogenous (wild-type) *Rlp22* is expressed. The group crossed RiboTag mice with several neuron-specific Cre recombinase-expressing mice. Brain tissues from the resulting offspring were used to immunopurify HA-tagged polysomes and recover associated mRNAs from specific cell populations expressing Cre recombinase. They demonstrated that the RiboTag system indeed reliably purifies mRNAs associated with ribosomes in a cell type specific manner.

The RiboTag strategy has several advantages over TRAP. First, the RiboTag mouse can be crossed to any Cre recombinase-expressing mouse line, allowing for polysome profiling of a significantly greater variety of cell types. In contrast, the TRAP system requires engineering a separate bacTRAP mouse line for each cell type of interest. Second, Rlp22^HA^ is expressed at levels similar to wild-type Rlp22, thereby maintaining the appropriate stoichiometry of ribosomal subunits and kinetics of translation. The TRAP system instead expresses the EGFP-L10a transgene from an exogenous promoter, which may result in different levels of EGFP-L10a compared to wild-type L10a. Third, the TRAP method recommends using in-house monoclonal antibodies against EGFP, which is costly for multiple experiments. Alternatively, the RiboTag method uses commercial anti-HA antibodies that are far more cost effective. Finally, RiboTag technology has the ability to generate Rlp22^HA^-expressing cells (e.g., Rpl22^HA^-expressing mouse embryo fibroblasts) that are capable of proliferating indefinitely in culture dishes, providing an abundant and renewable model for obtaining ribosome-associated mRNAs by immunoprecipitation rather than by the laborious process of sucrose gradient centrifugation. This ability is severely limited with the TRAP system since many of the current bacTRAP mice activate EGFP-L10a expression in neurons, which do not proliferate *in vitro*. Overall, the TRAP and RiboTag technologies provide an efficient and rapid method of isolating polysome-associated mRNA from a single cell type *in vivo*.

### Ribosome profiling

Many studies that have been designed to investigate global translation typically use genome-wide measurements of mRNA and/or protein expression as indicators of protein synthesis. However, transcripts are subject to multiple levels of translational control rendering mRNA expression an imperfect substitute for protein synthesis. Global protein expression, as measured by mass spectrometry, is also a poor proxy of translation since protein stability also contributes to changes in protein expression. The most precise measurement of translation is direct quantification of protein synthesis. To measure protein synthesis at a genome-wide level, Weissmann and colleagues developed a technique called ribosome profiling (Figure 2D) to map the precise positions of ribosomes within the transcriptome (Ingolia et al., 2009, 2012). Ribosome profiling involves nuclease digestion of cell extracts to degrade mRNA that is unprotected by ribosomes, leaving approximately 28 nucleotide RNA fragments. Individual ribosome-RNA complexes are isolated by centrifugation through a sucrose gradient or cushion, followed by a series of purification steps to recover the mRNA fragments, such as fragment size selection by gel electrophoresis and rRNA removal by subtractive hybridization. Ribosome-protected mRNA fragments (ribosome footprints) are identified by high-throughput sequencing, revealing the locations of ribosomes along transcripts at nucleotide-level resolution.

Ingolia et al. (2009) first demonstrated the utility of ribosome profiling for studying translation in *Saccharomyces cerevisiae*. The authors showed that ribosome profiling could quantitatively measure translational efficiency as defined by the ratio of ribosome footprint density to total mRNA. Ribosome density proved to be a much better predictor of protein production than measurements of mRNA levels. Ribosome density also correlated well, though not perfectly, with protein expression, suggesting that in some contexts mass spectrometry does not accurately measure global protein synthesis. The correlative differences between protein expression and translation efficiency observed by Ingolia et al. (2009) may be attributed to protein degradation and such differences could be exploited to investigate protein stability at a genome-wide level.

In addition to being an optimal tool for genome-wide measurements of protein synthesis, ribosome profiling is capable of discovering novel regulatory mechanisms of translation. While ribosome footprints are expected to map to coding regions of transcripts, a ribosome profiling study in yeast found that a small fraction of footprints (1.2%) mapped to non-coding regions (Ingolia et al., 2009). The majority of this 1.2% footprint fraction mapped to the 5′ UTRs of transcripts, leading to the discovery of 153 upstream open reading frames (uORFs) of which fewer than 30 had previously been described. Because ribosome profiling maps ribosome footprints at a resolution such that a three-base codon periodicity is observed, positional data can be used to identify frame shifts, non-canonical start codons, and stop codon readthrough, all of which would predict novel protein isoforms. As proof-of-principle, Ingolia et al. (2009) uncovered a total of 143 non-canonical (non-AUG) start sites in the yeast transcriptome; this was a substantial contribution to our knowledge of translational initiation sites in yeast, having previously known of only two examples of non-AUG initiation sites (Chang and Wang, 2004; Tang et al., 2004). Sites of premature translational termination can also be identified with this technique. Subsequent ribosome profiling studies have been implemented in other model organisms, including bacterial and mammalian systems (Guo et al., 2010; Ingolia et al., 2011; Oh et al., 2011; Hsieh et al., 2012). Not surprisingly, these studies also produced a long list of candidate initiation start sites, alternative reading frames, and uORFs. A comparison of these features across model systems or among different cell types will be informative in determining whether translational control mechanisms are general or specific to individual organisms and cell types.

Translation is a dynamic process and the kinetics of ribosome movement along transcripts is an important aspect of translational control. While the temporally static nature of ribosome profiling may appear ineffective for the study of translational kinetics, Ingolia et al. (2011) demonstrated otherwise by combining ribosome profiling with run-off elongation assays. First, cells were treated with harringtonine to block additional rounds of translation initiation (Fresno et al., 1977). Cycloheximide was then applied to cells for various time points afterward, freezing all actively translating ribosomes. Ribosome positions were then determined by ribosome profiling and the change in ribosome positioning over the course of cycloheximide treatment was used to generate a moving picture of ribosomes. In log phase growing yeast, the rate of translation appeared to be independent of mRNA class, mRNA length, or whether mRNAs encoded secreted or cytoplasmic proteins. This adaptation of ribosome profiling will prove useful in studying the rate of translation in the context of cellular stress or pathological states where global translation is aberrant (Shenton et al., 2006).

Another critical aspect of translational kinetics is ribosomal pausing. Pausing or stalling of elongating ribosomes is a mechanism of translational repression and may be caused by steric hindrance (due to secondary structure of the transcript or by the exiting nascent peptide), recruitment of low abundance tRNAs to rare codons, or RBPs such as FMRP (Lovett and Rogers, 1996; McNulty et al., 2003; Darnell et al., 2011). Ingolia et al. (2011) reasoned that the ribosome density for a given codon should be commensurate with the average ribosome dwell time, i.e., slower ribosome movement across a codon (longer dwell time) should result in more footprint counts at that codon. Based on this inference, the group used ribosome profiling to identify thousands of ribosome pause sites at the resolution of individual codons in the transcriptome of mouse embryonic stem cells (ESCs). Ribosomal pausing was initially described more than 20 years ago (Wolin and Walter, 1988), yet the mechanisms underlying this process is not well understood. Recently, ribosome profiling in bacteria was used to show that a Shine–Dalgarno-like feature in mRNA facilitates ribosome pausing (Li et al., 2012), demonstrating that this technique provides an efficient method to elucidate the molecular mechanisms of ribosomal pausing.

Similar to polysome profiling, ribosome profiling is technically challenging and labor intensive. Another consideration regarding ribosome profiling, as pointed out by Morris (2009), relates to the use of cycloheximide to halt elongating ribosomes prior to cell lysate preparation. Although characterized as an inhibitor of elongation, cycloheximide has also been shown to block translation initiation at similar concentrations used for ribosome profiling (Obrig et al., 1971) but not at lower concentrations (Lodish, 1971). If the exposure of cycloheximide to cells is low such that translation elongation, but not initiation, is negatively affected, this may allow ribosomes to accumulate at the 5′ end of transcripts and result in spurious ribosome footprinting patterns. This is especially relevant to applications of ribosome footprinting where drug delivery and exposure are difficult to control, for example with studies using mice. Yet despite these drawbacks, ribosome profiling remains a powerful tool to identify novel uORFs, initiation start sites, termination sites, and alternative reading frames, and provide insights on ribosome movement or lack thereof (ribosomal pausing) will most certainly lead to the discovery of new and unexpected modes of translational control.

### Targeting RBPs: Immunoprecipitation-based methods to isolate RBP-mRNA complexes

Key to understanding the role of RBPs in translational control, and RNA metabolism in general, is identifying their mRNA targets. Initial genome-wide attempts to identify RBP targets employed a technique called RNA immunoprecipitation (RIP). Much like its DNA counterpart chromatin immunoprecipitation, RIP involves formaldehyde crosslinking of proteins to RNA, followed by immunoprecipitation of the protein-RNA complex. Protein-bound transcripts are then used to make a cDNA library that is subjected to microarray analysis. A major concern with RIP is the low signal to noise ratio: RNA tends to be “sticky” making the technique vulnerable to extracting non-physiological binding partners (Mili and Steitz, 2004). The Darnell group introduced an alternate approach to RBP target identification termed CLIP for crosslinking and immunoprecipitation (Ule et al., 2003), which is reviewed in greater detail elsewhere (Darnell, 2010a; König et al., 2012). Instead of using formaldehyde, CLIP uses UV irradiation to cement protein-RNA interactions by creating covalent bonds between proteins and RNA that are within distances of a few angstroms. Unprotected RNA is removed by partial digestion with RNase (in the original protocol) or micrococcal nuclease, which is easily inactivated by EGTA to avoid spurious, continual RNA digestion throughout the procedure (Yeo et al., 2009; Zisoulis et al., 2010; Polymenidou et al., 2011). Protein-RNA complexes are recovered by immunoprecipitation with antibodies against the protein of interest. Transcript fragments of approximately 60–100 nucleotides in length are released from proteins and are further processed for sequencing. Several aspects of this technique make it well suited to study RBP-RNA interactions. First, the direct interaction between a RBP and its target mRNA is faithfully preserved, since crosslinking only occurs with RBPs and mRNA that are within angstrom distances. Second, crosslinking of direct RBP-mRNA interactions via strong covalent bonds allows these complexes to be purified under stringent conditions, further reducing background signal. Third, UV irradiation does not preserve protein–protein interactions, thereby avoiding the possibility of indirect protein-mRNA interactions.

The first application of CLIP sought to identify RNA targets of Nova, a neuronal KH-type RBP, which is implicated in paraneoplastic neurologic degeneration (Darnell, 2010b). In this study, Ule, Jensen, and colleagues identified 34 candidate mRNA targets, most of which are involved in neuron function (Ule et al., 2003). The use of low-throughput sequencing to identify Nova targets, however, generated a limited dataset and made it difficult to discern authentic from spurious mRNA targets (50% false positive rate). To remedy this, the same group performed a subsequent study in which CLIP was combined with high-throughput sequencing (HITS-CLIP). This strategy generated a more robust dataset of Nova targets, confirming, and refining their previous assertions about Nova as a splicing regulator (Licatalosi et al., 2008). Further modifications to the standard CLIP protocol have improved the crosslinking efficiency. One such modification called Photoactivatable-Ribonucleoside-Enhanced Crosslinking and Immunoprecipitation, or PAR-CLIP, uses photoreactive ribonucleoside analogs [e.g., 4-thiouridine (4SU) or 6-thioguanosine], which are incorporated into nascent mRNAs in live cells (Hafner et al., 2010) or whole organisms (Jungkamp et al., 2011). Photoreactive ribonucleoside analogs crosslink with proteins more efficiently than endogenous ribonucleotides, thereby enhancing the signal to noise ratio. During cDNA preparation of labeled mRNA, crosslinked sites are prone to thymidine to cytidine or guanosine to adenosine transitions (when 4-thiouridine or 6-thioguanosine is used, respectively), revealing exact locations of nucleotide-protein interactions. This feature was initially exploited to identify individual ribonucleotides of a small nucleolar RNA that interacted with RBPs (Granneman et al., 2009) and has since been applied to other RBPs (Hafner et al., 2010). It is important to note that not all RBPs may be amenable to PAR-CLIP, such as CUG triplet repeat RNA binding protein (CELF1; Castello et al., 2012). A further modification of CLIP, called individual nucleotide resolution CLIP or iCLIP, provided an alternative approach to locating the exact crosslinking position (König et al., 2010). Ule and colleagues took advantage of the fact that reverse transcriptase arrests at sites of nucleotide-peptide crosslinking (a peptide remnant of the RBP remains after proteinase K digestion). The resulting truncated cDNAs are normally lost during the standard CLIP library preparation, but the iCLIP protocol was designed to recover and sequence these truncated cDNAs to identify exact crosslinking sites. Using CLIP or its variants, genome-wide protein-RNA interaction maps have been assembled for numerous RBPs, including Nova (Ule et al., 2003), RBFOX2 (Yeo et al., 2009), Argonaute proteins (Chi et al., 2009; Hafner et al., 2010; Zisoulis et al., 2010; Leung et al., 2011), TDP-43 (Polymenidou et al., 2011; Tollervey et al., 2011), FMRP (Darnell et al., 2011), and hnRNP proteins (Katz et al., 2010; König et al., 2010; Huelga et al., 2012).

The ability to systematically uncover RBP target sites within the transcriptome has provided insights to the role of RBPs in translational control. Using *in vivo* PAR-CLIP (iPAR-CLIP), Jungkamp et al. (2011) proposed several models by which GLD-1, a conserved germline-specific RBP in *C. elegans*, functioned as a translational repressor. Highly conserved 5′UTR GLD-1 binding sites were discovered near the start codon of target transcripts; this was unexpected, since prior studies indicated that GLD-1 primarily targets the 3′ UTR. Following extensive biochemical validation of their iPAR-CLIP results, Jungcamp and colleagues proposed that GLD-1 dimers, as the protein is known to form, bind to the 5′ and 3′ UTRs to promote circularization of mRNA to block translational initiation (Jungkamp et al., 2011). Alternatively, binding of GLD-1 near start codons at the 5′ UTR may prevent assembly of the ribosome.

Another study examined the mechanisms of translational control by FMRP using CLIP. Darnell et al. (2011) identified FMRP binding sites within the polysome-associated fraction of the transcriptome by HITS-CLIP and found that a significant portion of gene targets were involved in neuronal synaptic plasticity and synaptic-related signaling pathways. The majority of FMRP binding sites (66%) resided within coding sequences. This was unexpected given that translational control mechanisms usually involve binding of RBPs within UTRs. Furthermore, the distribution of FMRP appeared to be uniform along transcripts. Darnell and colleagues extended these observations to demonstrate that FMRP directly stalls ribosomes on polysome-associated transcripts, thereby suppressing translation. Given that many of these FMRP targets are involved in synaptic transmission, it was hypothesized that FMRP functions to represses translation of associated mRNAs during transit from the soma to synapses, to prevent premature translation and/or degradation. Upon release of FMRP from its targets, presumably by signal activation, the transcripts, already loaded with ribosomes, would be rapidly translated. Given that the interplay between different RBPs is important for RNA transport and local synaptic translation, the application of CLIP to neuronal RBPs will be instrumental in defining their individual and combinatorial contributions to translational control.

CLIP is accompanied by limitations that are either unique to particular version or inherent to all (Darnell, 2010a; Ascano et al., 2012). The ability of CLIP to capture a representative population of RNA targets is influenced by the crosslinking efficiency between RNA and the protein of interest, nuclease digestion conditions (Kishore et al., 2011), and the specificity and reactivity of the antibody used to isolate the RBP-RNA complex; these factors are inherent to all CLIP versions. Crosslinking efficiency will vary depending on the RNA target sequence and the type of amino acids available for crosslinking. Nucleic acids are generally more reactive with cysteine, lysine, phenylalanine, tryptophan, and tyrosine residues, and this residue preference appears to be preserved with 4SU-labeled RNA (Meisenheimer and Koch, 1997; Meisenheimer et al., 2000). 4SU is generally thought to enhance crosslinking efficiency compared to unmodified uridine (Hafner et al., 2010), although this is not always the case (Kishore et al., 2011). A drawback of PAR-CLIP is the difficulty in applying this method to whole animal models other than *C. elegans*, where uniform exposure of 4SU may not be feasible. Until a method for efficient delivery of ribonucleoside analogs to other animal models (namely mice) is established, HITS-CLIP is generally required for *in vivo* studies.

## Challenges and Considerations for Genome-Wide Studies in Neurons

The application of genome-wide studies to neurobiology is accompanied by challenges intrinsic to the neural model system and genome-wide method being employed. The mammalian CNS is complex with hundreds of morphologically distinct cell types, each expressing a unique transcriptome. This presents several challenges when performing genome-wide studies: purifying homogenous cell populations, isolating cells in a manner that does not alter gene expression, and obtaining sufficient quantities of cells for analysis. With regard to the latter issue, some of the techniques discussed here require up to several tens of micrograms of RNA for analysis, necessitating a large input of cells. Cultured primary neurons derived from rodent tissue are a well established model for studying neurons; however, they do not obviate some of these challenges, as they may not be completely homogenous and do not expand *in vitro*. Furthermore, establishing and maintaining cultured primary neurons are difficult and require extensive training of the researcher. Neural progenitor cells (NPCs), either differentiated from stem cells or isolated from rodents or humans, are an alternative *in vitro* model system. NPCs can be expanded and differentiated into multiple neuronal cell types, and developing an efficient differentiation process should yield a fairly homogenous cell population. Importantly, NPCs that were differentiated from induced pluripotent stem cells (iPSCs) derived from patient fibroblasts have been shown to recapitulate the molecular phenotypes of corresponding neurological diseases (Thonhoff et al., 2009; Ming et al., 2011), providing valuable models to investigate global translation in neurodegeneration using the methods described herein. The labor intensive, lengthy, and costly nature of establishing iPSCs and differentiating them into NPCs is a major drawback of this system.

Similar to cell culture models, animal models used for genome-wide analyses are fraught with the challenges mentioned above; however, some of these challenges may be circumvent with the use current technologies. To remedy the issue of complex cell heterogeneity, the RiboTag and TRAP technologies were created, both of which expressed tagged versions of ribosomes in a cell type specific manner to isolate mRNA. While the intended application of RiboTag and TRAP technologies is for *in vivo* polysome profiling, conceivably they may be adapted for *in vivo* ribosome profiling studies. The application of HITS-CLIP in heterogenic mouse brain tissue is standard (Licatalosi et al., 2008; Polymenidou et al., 2011), and in theory the technology required to perform HITS-CLIP in a cell type specific manner *in vivo* is obvious. Using transgenic mice that express a tagged version of the RBP of interest in a cell type specific manner, HITS-CLIP could easily be implemented *in vivo* using an antibody that recognizes the tag. Naturally, adaptation of RiboTag or TRAP technologies for ribosome profiling or transgenic mice for HITS-CLIP will require optimization steps to ensure a seamless integration.

Lastly, bioinformatic challenges inherent to genome-wide studies largely center on the issue of interpreting the massive amounts of raw sequencing data. A convergent problem in analyzing sequence reads derived from CLIP and ribosome profiling data is the identification of precise binding sites of RBPs or ribosome footprints within the RNA transcripts. Thus far, the analysis of CLIP data relies on inherent assumptions about the binding kinetics of RBPs with their preferred RNA substrates. The RBPs that have been studied thus far, for example Nova, RBFOX2, Argonaute, and hnRNP family members (Ule et al., 2003; Yeo et al., 2009; Hafner et al., 2010; Zisoulis et al., 2010; Leung et al., 2011; Huelga et al., 2012), tend to interact with several binding sites within a given substrate very strongly. Computational approaches to identify precise binding sites (or clusters of reads), known as cluster- or peak-finding algorithms, have therefore assumed that the majority of reads within an RNA that are below an expected threshold are not true binding sites and represent experimental noise or artifacts. However, other RBPs, such as TDP-43 or FUS/TLS may act in “scanning” mode on some RNA substrates (unpublished observations), interacting with many sites with low affinity, rather than a few sites with high affinity, with nevertheless important biological effects. Most algorithms are conservative in identifying these low affinity sites within RNA substrates. Ribosome footprints fall into this second category of many locations of roughly equal occupancy with mRNA substrates. Thus while ribosome profiling data can be used to measure quantitative differences in ribosome occupancy within RNAs, precise footprint sites such as paused ribosomes are not effectively identified by existing peak-finding algorithms. Another glaring concern with analyzing RNA and ribosome binding data is the dearth of statistical tools to measure whether we have saturated the potential number of binding sites observed (one strategy is suggested in Polymenidou et al., 2011), or techniques to measure the rate of false positives and negatives. Lastly, not all RNA binding sites are functional. It is not unthinkable that while highly expressed RBPs interact with many RNA substrates, many, if not most of these sites may not actually have an impact in regulating the life cycle of the RNA molecule. Integrating other genome-wide assays that reveal the dependence of the RNA on the RBP is important for assigning functions to these binding sites. Thus, computational approaches to integrate these information with CLIP data, and also comparing how multiple RBPs affect the same molecules will be crucial in going forward (Huelga et al., 2012).

## RNA Binding Proteins and Translational Control in Neurological Diseases

RNA binding proteins have received considerable attention for their roles in neurodegeneration (Lukong et al., 2008; Liu-Yesucevitz et al., 2011). This is not surprising given that many RNA processing events, including local translation, are important for neuronal function (Klann and Dever, 2004; Sutton and Schuman, 2006). Of the handful of RBPs that are associated with neurological diseases, only several have been implicated in the regulation of translation (Table 2), most of which were determined by single-gene studies. Presently, there is a lack of genome-wide studies examining the translation functions of RBPs in normal or pathological contexts of neurons, with the exception of a few studies related to FMRP; this leaves much room for investigation. In this section, we review our current knowledge of these RBPs (listed in Table 2) with regard to translational control and neurological diseases.

**Table 2:** **List of RBPs involved in translation and implicated in neurological diseases**.

RBP	Function	Disease	Reference
FMRP	Repressor	FXS	Darnell et al. (2011)
hnRNP A2/B1	Activator	ALS, FTLD	Kwon et al. (1999)
hnRNP C	Activator	AD	Lee et al. (2010)
IGHMBP2	Regulator	SMA	Grohmann et al. (2001), de Planell-Saguer et al. (2009)
Musashi	Repressor	AD	Okano et al. (2002), Perry et al. (2012)
SMN	Putative repressor	SMA	Piazzon et al. (2008)
TDP-43	Repressor	ALS, FTLD	Lagier-Tourenne et al. (2010)

*AD, Alzheimer’s disease; ALS, Amyotrophic lateral sclerosis; FTLD, Frontotemporal lobar dementia; FXS, Fragile X syndrome; SMA, Spinal muscular atrophy*.

FMRP is one of the more extensively studied RBPs with regard to translational regulation and neurodegeneration. It is essential for proper synaptic function, as loss of FMRP in mice results in aberrant pre- and post-synaptic plasticity (Deng et al., 2011). FMRP is present throughout the neuron (soma, dendrites, and axon) with the majority of FMRP (85–90%) being associated with polysomes (Zhang and Darnell, 2011). Misregulation of FMRP is linked with FXS, a condition characterized by impaired cognitive, physical, emotional, and sensory function (Bagni and Greenough, 2005; Bassell and Warren, 2008). Consistent with this, a genome-wide analysis of FMRP RNA targets using HITS-CLIP revealed that many FMRP targets encode proteins that are implicated in autism spectrum disorders (Darnell et al., 2011). Mutations within the FMRP gene that lead to reduced expression are frequently observed in patients with FXS (De Boulle et al., 1993; Snow et al., 1993). As discussed in this review, FMRP downregulates target gene expression by blocking translation in part by stalling ribosomes. FMRP has also been shown to interact with Ago2 and recruit miRISCs to its mRNA targets, providing another mechanism by which FMRP mediates gene silencing (Muddashetty et al., 2011). Edbauer et al. (2010) demonstrated that FMRP associates with miR-125b and miR-132 in the mouse brain, interactions of which greatly influence dendritic spine morphology and synaptic physiology of hippocampal neurons. In this study, a limited set of miRNAs was examined and it is likely that additional miRNAs interact with FMRP. Genome-wide methods tailored to studying miRNA-protein interactions are well established (reviewed in Wilbert and Yeo, 2011) and would provide an unbiased approach toward identifying miRNAs that interact with FMRP as well as other RBPs.

The evolutionarily conserved Musashi family of RBPs, herein referred to as Musashi, has strong links to both translational control and neurobiology (Okano et al., 2002). Preferentially expressed in the mammalian nervous system, Musashi is a key contributor to maintaining the proliferative capacity of neural stem cells, such that its loss leads to a reduction in the formation of neurospheres, or cell-cultured neural stem cells (Sakakibara et al., 2002). Musashi is thought to maintain progenitor self-renewal by translationally repressing inhibitors of stem cell proliferation. One of these targets, *CDKN1A*, encodes the anti-proliferative cell-cycle inhibitor p21WAF (Battelli et al., 2006). *NUMB* is another Musashi target that is a negative regulator of the Notch signaling pathway. Since Notch signaling is crucial for neural stem cell maintenance (Hitoshi et al., 2002), translational silencing of *NUMB* by Musashi augments this proliferative process (Imai et al., 2001). The mechanism by which Musashi suppresses translation, at least for *NUMB*, involves disrupting the eIF4G binding-Poly(A)-binding protein interaction, thereby preventing assembly of the 80S ribosomal complex (Kawahara et al., 2008). If Musashi is characteristic of most RBPs, then it has hundreds or thousands targets in addition to *NUMB* and *CDKN1A*. Using the iterative *in vitro* selection process SELEX, the RNA binding sequence of Musashi has been identified (Imai et al., 2001). This sequence information can be useful in predicting targets, yet still requires single-gene studies to validate authentic Musashi targets. The application of HITS-CLIP to define Musashi targets and locate discrete binding sites would prove invaluable toward understanding the function of Musashi. This knowledge may also be useful to investigate a potential role of Musashi in Alzheimer’s disease (AD). A link between Musashi and AD was established with the observation that Musashi expression was reduced in patients with AD (Perry et al., 2012). Consistent with Musashi being a key regulator of neural stem cells, downregulation of Musashi correlated with a reduction in neural stem cells, the latter event of which is often observed in AD. Whether misexpression of Musashi is a determinant or downstream effect of AD is uncertain. As will be a common theme among the RBPs addressed in this section, a comprehensive understanding of Musashi function as revealed by genome-wide studies will likely identify therapeutic targets implicated in AD.

TAR DNA-binding protein-43 (TDP-43) is another RBP that has recently been recognized as an important contributor to neurological diseases (Lagier-Tourenne et al., 2010). It is well established as a regulator of transcriptional repression and alternative splicing, but may also have a role in translational repression (Wang et al., 2008c). In hippocampal neurons, TDP-43 appears to reside within RNA granules and P-bodies – storage sites of repressed mRNAs; this observation is consistent with the finding that TDP-43 acts as a translational repressor in an *in vitro* assay (Wang et al., 2008b). A pathological link between TDP-43 and neurodegeneration was initially established with the observation that TDP-43 is present within brain cell inclusions of patients with ALS or FTLD (Arai et al., 2006; Neumann et al., 2006). Dominant mutations in the gene encoding TDP-43 (*TARDBP*) that cause mislocalization of the protein were subsequently identified in cohorts of patients with ALS and FTLD (Gitcho et al., 2008; Kabashi et al., 2008; Sreedharan et al., 2008; Van Deerlin et al., 2008; Yokoseki et al., 2008). The aberrant activities of disease-associated forms of TDP-43 have been recapitulated in transgenic rats displaying neurological impairments and in reprogrammed pluripotent stem cells, confirming the pathogenicity of these mutations (Bilican et al., 2012; Huang et al., 2012). Findings from genome-wide studies have advanced our understanding of the pathogenic activities TDP-43 by revealing that TDP-43 modulates the levels and alternative splicing of many of its RNA targets, a substantial portion of which encode proteins involved in neuronal development and function (Polymenidou et al., 2011; Tollervey et al., 2011). A connection between the translational silencing and alternative splicing functions of TDP-43 may exist, as demonstrated for the TDP-43 target S6 kinase 1 (S6K1) Aly/REF-like target (SKAR). TDP-43 appears to control alternative splicing of SKAR, which results in the expression of a SKAR isoform that can no longer activate the translation-stimulating SK61-dependent signaling pathway (Fiesel et al., 2012). Therefore loss of TDP-43 resulted in upregulation of the SKAR isoform that increases SK61 activity and consequently stimulated global translation. Whether the translational functions of TDP-43 require its splicing activities for other TDP-43 targets remains elusive. But more pressingly, the role of TDP-43 in global translation remains unknown and will best be addressed by employing the genome-wide methods discussed here.

Two members of the heterogeneous nuclear ribonucleoproteins (hnRNPs) family of RBPs, hnRNP A2/B1, and hnRNP C, have been recognized as translational regulators with links to neurological diseases. As key regulators of RNA metabolism, hnRNP proteins are widely expressed in various tissues, including the mammalian brain (Kamma et al., 1995, 1999). In neuroblastoma cells, hnRNP C was shown to enhance the translation of mRNA that encode amyloid precursor protein (APP), a protein that, when aberrantly processed, is the major constituent of cerebral amyloid plaques found in patients with AD (Lee et al., 2010). By associating with the same region of APP mRNA as FMRP, hnRNP C prevents FMRP from binding to and silencing the translation of APP mRNA. Another hnRNP family member, hnRNP A2/B1, promotes translation through several mechanisms. It has been show to mediate transport of specific mRNAs to distant dendrites where they are translated to produce a local supply of proteins that are required for synaptic plasticity (Gao et al., 2008). HnRNP A2/B1 was also shown to control the translation of mRNAs encoding myelin basic protein and c-Myc through mechanisms that are not well defined (Kwon et al., 1999; Shi et al., 2011). Similar to TDP-43, hnRNP A2/B1 is present within brain cell inclusions of some patients with FXTAS, suggesting that the pathogenic functions of hnRNP A2/B1 involve its mislocalization (Iwahashi et al., 2006).

Several other neurological disease-related RBPs are suggested to have roles in translation, yet direct experimental evidence to support such roles is lacking. One example is survival of motor neuron (SMN), an RBP that is mutated in patients with SMA (Wirth, 2000). SMA is a fatal autosomal recessive disorder characterized by degeneration of lower motor neurons that ultimately results in paralysis with muscular atrophy (Lunn and Wang, 2008). SMN is involved in the assembly of the spliceosome, a complex that carries out gene splicing, and is also required in the formation of SGs (Liu et al., 1997; Pellizzoni et al., 1998; Hua and Zhou, 2004). In addition, SMN is found in complexes with FMRP, and through this association, SMN is hypothesized to mediate translation (Piazzon et al., 2008). Loss-of-function mutations in the gene encoding the RBP immunoglobulin μ-binding protein 2 (IGHMBP2) are also associated with SMA (Grohmann et al., 2001). Biochemical analysis of IGHMBP2 determined that it physically associates with regulators of tRNA transcription and ribosome biogenesis, suggesting strong ties to translational regulation (de Planell-Saguer et al., 2009). The mechanism by which defects in SMN and IGHMBP2 cause SMA or whether either protein has a defined role in translation remains unknown.

## Conclusion

Although progress has been made in understanding translational regulation in neurons, many questions remain unaddressed. Specifically, our knowledge of RBP function in translation is very limited. We have yet to discover direct targets of many neuron-related RBPs and determine how these RBPs influence ribosome kinetics and the distribution of mRNAs with different mRNP complexes. We also do not know whether misregulation of certain RBPs, an event often associated with neurological diseases, affects global translation and, if so, whether aberrant translational regulation is an underlying mechanism leading to disease. Genome-wide methods are well suited to address these questions, and can provide highly detailed information that will reveal novel translational control mechanisms. The tool set of genome-wide methods to study translational control is extensive, and improvements to both the molecular and computational components of these techniques are ongoing. Since genome-wide studies have the capacity to produce an overwhelming amount of data, it is important that key findings be independently validated by molecular techniques. Such complementation between genome-wide and molecular studies will undoubtedly provide insights to the basis for translational regulation in neurons.

## Conflict of Interest Statement

The authors declare that the research was conducted in the absence of any commercial or financial relationships that could be construed as a potential conflict of interest.
